# Perioperative low molecular weight heparin bridging in aortic mechanical heart valve patients undergoing endoscopic procedures

**DOI:** 10.1055/a-2840-7302

**Published:** 2026-04-17

**Authors:** Tegvir Singh Grewal, Alejandro Daniel Godoy, Vinai Bhagirath, Ana I Casanegra, Alfonso Tafur, Amelia McNiven Fontani, Atefeh Ghorbanzadeh, Damon E Houghton, Jameel Abdulrehman, Jean-Philippe Galanaud, Luigi D Sordo, Mouza Alnuaimi, Paul R Daniels, Stephanie Carlin, Yama Sadozai, Alan Barkun, James Douketis

**Affiliations:** 1 33493 Department of Biomedical Sciences, Population Health Research Institute, Hamilton, Canada; 2 4257 School of Medicine, Queen's University, Kingston, Canada; 3 33493 Thrombosis, Population Health Research Institute, Hamilton, Canada; 4 Thrombosis, Hamilton Health Sciences Corporation, Hamilton, Ontario, Canada; 5 3710 Thrombosis, McMaster University, Hamilton, Canada; 6 6915 Gonda Vascular Center, Mayo Clinic, Rochester, United States; 7 3271 Vascular Medicine, Endeavor Health, Evanston, United States; 8 7938 University Health Network, University of Toronto, Toronto, Canada; 9 71545 Thrombosis, Sunnybrook Health Sciences Centre, Toronto, Canada; 10 3710 Internal Medicine, McMaster University, Hamilton, Canada; 11 Division of Gastroenterology, Rm D7 148, Montreal General Hospital, Montreal, Canada; 12 Thrombosis, St. Joseph's Healthcare Hamilton, Hamilton, Ontario, Canada

**Keywords:** Endoscopy Upper GI Tract, Non-variceal bleeding, Endoscopy Lower GI Tract, Polyps / adenomas / ...

## Abstract

**Background and study aims:**

Optimal management of anticoagulation for patients with aortic mechanical heart valves (MHVs) receiving vitamin K antagonists (VKAs) undergoing gastrointestinal endoscopic procedures is clinically challenging. Risk of bleeding from an unanticipated polypectomy or biopsy further complicates this issue. Current guidelines on use of bridging with low-molecular-weight heparin (LMWH) for these procedures are based on low-quality evidence.

**Patients and methods:**

We conducted a subanalysis of a retrospective, multicenter observational study of adult patients with aortic MHVs receiving VKAs who underwent elective gastrointestinal endoscopies (colonoscopy or upper endoscopy). We included patients who underwent the procedure between July 1, 2020, and July 1, 2023, at five centers in Canada and the United States Patients with procedures performed on separate days within a 30-day period were excluded. Efficacy and safety outcomes included thromboembolic events, major bleeding (MB), and clinically relevant non-major bleeding (CRNMB) in the 30 days post procedure.

**Results:**

A total of 192 endoscopic procedures were analyzed. Median (interquartile range [IQR]) patient age was 66 years (58.2–72.8) and 22% were female. Warfarin was interrupted for 185 procedures (96%). Among these, 119 (64%) received LMWH bridging: 33 (17%) pre-procedure only, 10 (5%) post-procedure only, and 76 (40%) both pre- and post-procedure. Post-procedure LMWH was initiated a median (IQR) of 1 day (1–1) and discontinued 5 days post-procedure (3–8). MB occurred in two patients (1%), CRNMB in two patients (1%), and one death (0.5%) was identified; no thromboembolic events occurred. Prior to propensity score matching (PSM), post-procedure LMWH with or without pre procedure LMWH was associated with the composite outcome of MB and CRNMB (P = 0.034), although not with MB or CRNMB individually. After stepwise regression and PSM analyses, no significant differences in bleeding outcomes were observed between patients who received LMWH bridging and those who did not.

**Conclusions:**

Our findings indicate that although adverse thrombotic events were rare and use of post-procedure LMWH bridging was significantly associated with a small absolute increased risk of bleeding, it was not significant after PSM. Larger prospective studies are needed to better inform antithrombotic management guidelines for patients with aortic MHVs undergoing gastrointestinal endoscopy.

## Introduction

Patients with mechanical heart valves (MHVs) require lifelong anticoagulation with vitamin K antagonists (VKAs) to prevent thromboembolic complications due to the thrombogenic nature of artificial valves 1 . Temporary discontinuation of VKAs is necessary to maintain hemostasis during invasive procedures but this interruption may heighten risk of thromboembolism 1 . In light of the updated U.S. Preventive Services Task Force (USPSTF) guidelines recommending colorectal cancer screening for all adults aged 45 to 75 years, the number of endoscopic procedures necessitating interruption of VKA therapy is increasing 2 . This trend represents a growing perioperative management challenge in patients with MHVs, of whom approximately 13% will require invasive procedures annually 1 .

Anticoagulant bridging with low-molecular-weight heparin (LMWH) during the interruption of VKAs can be administered to reduce duration of anticoagulation interruption with the aim of reducing risk of thromboembolism during the periprocedural period 1 . However, LMWH bridging may increase risk of bleeding, especially if there is residual anticoagulant effect at the time of, or shortly after, a procedure 1 . Previous studies have demonstrated a lower risk of thrombosis with bileaflet aortic MHVs, leading to less bridging in these patients 3 .

Data to guide periprocedural anticoagulation management in MHV patients undergoing gastrointestinal endoscopic procedures are limited. Although recent guidelines from the British Society of Gastroenterology and European Society of Gastrointestinal Endoscopy provide recommendations based on procedural bleeding risk, bleeding risk associated with endoscopy is variable and less predictable 4 . Thus, a seemingly low-risk diagnostic endoscopy can become high-risk for bleeding if a polyp removal is required 5 . Post-procedural anticoagulation may also require adjustment, including omission of post-procedural LMWH or use of a prophylactic-, rather than therapeutic-dose regimen 5 . Additional data to establish optimal periprocedural anticoagulation management in MHV patients undergoing gastrointestinal endoscopies, therefore, are required.

The AMBER (Aortic Mechanical Valve Bridging Evaluation in Surgery) study reported that LMWH bridging in aortic MHV patients undergoing elective procedures was associated with increased bleeding without a reduction in thromboembolic events 6 . However, outcomes for patients undergoing gastrointestinal endoscopy, a particularly common procedure, have not been described. In this subanalysis of the AMBER cohort, we aimed to characterize rates of bleeding and thromboembolic complications in patients with aortic MHVs undergoing gastrointestinal endoscopic procedures, comparing outcomes between patients managed with and without LMWH bridging.

## Patients and methods

### Study design

AMBER was a retrospective, multicenter study investigating perioperative bleeding and thromboembolic outcomes of patients with aortic MHV and VKA interruption for an elective procedure/surgery. The study was conducted at five academic centers in Canada and the United States (Hamilton Health Sciences, Hamilton, ON, St. Joseph’s Healthcare Hamilton, Hamilton ON, Mayo Clinic, Rochester MN, Endeavor Health System, Evanston Illinois, United States, Sunnybrook Health Sciences Center, Toronto ON) and was approved by the institutional review boards at all sites.

### Patients

Consecutive eligible patients were identified from electronic medical records between July 1, 2020, to July 1, 2023, and patient data were input into case report forms in REDCAP systems 7 . Patients were identified by searching perioperative assessment clinics, anticoagulation management clinics and perioperative anticoagulation/bridging clinic records, to compare patients who received or did not receive periprocedural LMWH bridging.

Patients were included if they satisfied the following criteria: 1) age ≥ 18 years; 2) bileaflet aortic MHV; 3) elective gastrointestinal endoscopy procedure (if patients had multiple procedures, they must have occurred on the same day or at least 30 days apart); and 4) clinical follow-up documentation for at least 30 days post-procedure. Information about polyp removal or biopsy was collected from procedure reports. Patients were excluded if they had: 1) mitral and aortic MHVs; or 2) multiple gastrointestinal endoscopic procedures during the 30-day post-procedure observation period.

### Definitions of periprocedural heparin bridging

LMWH bridging was defined based on The American College of Chest Physicians clinical practice guideline on perioperative anticoagulant management, comprising: enoxaparin 1 mg/kg bid or 1.5 mg/kg daily; dalteparin 100 IU/kg bid or 200 IU/kg daily, or tinzaparin 175 IU/kg daily 8 . Bridging includes any perioperative administration of LMWH and was classified as pre- and post-procedure, only pre-procedure, or only post-procedure 8 .

### Study outcomes

The primary safety outcome was clinically relevant non-major bleeding (CRNMB) or major bleeding (MB), as defined by the International Society on Thrombosis and Haemostasis, within 30 days post-procedure 9 . A secondary safety outcome was clinically relevant bleeding, defined as the composite of CRNMB and MB. The primary efficacy outcome was a composite of arterial thromboembolism, comprising ischemic stroke, myocardial infarction and systemic embolism and venous thromboembolism, comprising pulmonary embolism and deep vein thrombosis. All outcomes were such that they required a hospital-based assessment, to be captured in site-based medical records. Outcomes occurring within 30 days post-procedure were included in this study and adjudicated by the principal investigator at each site.

### Statistical analysis

The analysis was performed per procedure because several patients had multiple procedures during the study period, and all data reflect findings for a single procedure. Descriptive and comparative statistical analyses were performed to examine differences between patients based on the LMWH bridging regimen received. Categorical variables were compared using the chi-square or Fisher’s exact test, and odds ratios (ORs). Non-normally distributed continuous variables were compared using non-parametric tests (Mann-Whitney U).

Logistic regression analysis was performed to evaluate the effect of LMWH bridging and other variables on bleeding outcomes; no regression analysis was done for thromboembolic outcomes due to insufficient events. Multivariate logistic regression, using a stepwise selection method, was done to determine covariate combinations independently associated with bleeding outcomes.

To further analyze these relationships, sensitivity analyses were through propensity score matching (PSM) to compare bleeding outcomes among groups matched based on baseline patient characteristics. The effect of pre- and post-procedure bridging was evaluated as compared with no bridging. A propensity score estimation was completed for each patient based on covariates associated with bleeding outcomes from the entire AMBER cohort: age, hypertension; chronic kidney disease, atrial fibrillation, CHADS2 score, years since valve replacement, and doubling of initial warfarin dose post-procedure. The nearest neighborhood matching method was used, with a 1:1 ratio, using a caliper width of 0.2 standard deviation of the logit of the propensity score.

## Results

### Patient characteristics

Baseline patient characteristics are shown in Table 1 . Median age was 66 years (interquartile range [IQR] 58–73) and 43 patients (22%) were female. Atrial fibrillation was present in 41% of patients, 14% had history of stroke or transient ischemic attack, and 7% had venous thromboembolism.

**Table:** **Table 1** Baseline patient characteristics.

**Patient characteristic**	
Age at time of procedure (years, IQR)	66 (58.2–72.8)
Female n (%)	43 (22.4%)
Weight (kg, IQR)	87.8 (77.1–103.9)
Center	
Hamilton General Hospital	53 (27.6%)
St. Joesph’s Healthcare	40 (20.8%)
Mayo Clinic	73 (38.0%)
Sunnybrook	7 (3.65%)
NorthShore University Health System	19 (9.90%)
Diabetes	48 (25.0%)
Hypertension	139 (72.4%)
Congestive heart failure	54 (28.1%)
Coronary artery disease	72 (37.5%)
Peripheral artery disease	16 (8.33%)
History of revascularization surgery for either CAD or PAD	54 (28.1%)
Chronic Kidney Disease	31 (16.1%)
Dialysis	20 (93.5%)
Type of valve	
Bileaflet	105 (54.7%)
Carbomedics	33/105 (31.4%)
St. Jude	21/105 (20.0%)
On-X	44/105 (23.5%)
Medtronic ATS	7/105 (3.74%)
Unknown	87 (45.3%)
Years since AMHV replacement (IQR)	15.3 (8.60–44.3)
History of atrial fibrillation	79 (41.1%)
Paroxysmal atrial fibrillation	29/79 (36.7%)
Persistent atrial fibrillation	15/79 (19.0%)
Other	6/79 (7.59%)
Not specified	29/79 (36.7%)
History of stroke/TIA	27 (14.1%)
History of venous thromboembolism	14 (7.29%)
Antiplatelet drug Use	76 (39.6%)
Values given as n (%) unless stated otherwise. AMHV, aortic mechanical heart valve; CAD, coronary artery disease; IQR, interquartile range; PAD, peripheral artery disease; TIA, transient ischemic attack.	

**Fig. 1:**
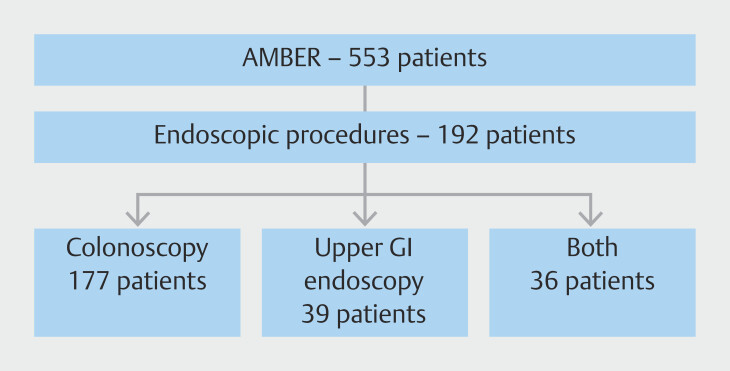
Subanalysis design overview. Patients were excluded if procedures occurred over several days in a 30-day period.

A total of 192 procedures were included ( Fig. 1 ): 153 colonoscopies (80%) and 39 upper gastrointestinal endoscopies (20%). With respect to additional interventions, polypectomies were most commonly performed (n = 77), followed by upper gastrointestinal endoscopy biopsies (n = 17) and colonoscopy biopsies (n = 5).

In addition to a VKA, 76 patients (40%) were taking aspirin. Periprocedural LMWH bridging therapy was administered to 119 patients (62%) and 185 patients (96%) interrupted warfarin use. As shown in Table 2, 33 patients (17%) received pre-procedure LMWH only, 10 (5%) received post-procedure LMWH bridging only, 76 patients (40%) received pre- and post-procedure LMWH, and 73 patients (38%) received no LMWH. Procedures in which a biopsy or polypectomy were performed are also detailed in Table 2 . Three (18%) of the 17 patients who had an upper gastrointestinal biopsy only received pre-procedure LMWH, seven (41%) of the 17 patients had biopsies with pre- and post-procedure bridging, six patients (35%) received no bridging, and one patient (6%) received only post-procedure bridging (6%). Of the 77 patients who had a polypectomy, 23 (25%) only received pre-procedure LMWH, 14 (15%) received pre and post procedure LMWH, 39 patients (43%) received no bridging, one patient (1%) received only post-procedure LMWH bridging. Only one patient (20%) of the five who had a colonoscopy and biopsy received LMWH pre- and post-procedure, and four patients (80%) did not receive any bridging.

**Table:** **Table 2** Periprocedural anticoagulant bridging.

	**LMWH pre-procedure (n = 33)**	**LMWH post procedure (n = 10)**	**LMWH pre- and post-procedure (n = 76)**	**No LMWH (n = 73)**
Upper gastrointestinal endoscopy (n = 39)				
Biopsy (n = 17)	3 (9.1%)	1 (10%)	7 (9.2%)	6 (8.2%)
No biopsy (n = 20)	1 (3.0%)	5 (50%)	12 (15.8%)	4 (5.5%)
Colonoscopy (n = 153)				
Polypectomy +/- biopsy (n = 77)	23 (69.7%)	1 (10%)	14 (18.4%)	39 (53.4%)
Biopsy (n = 5)	0 (0.0%)	0 (0.0%)	1 (1.32%)	4 (5.5%)
No biopsy or polypectomy (n = 73)	6 (18.2%)	3 (30%)	42 (55.3%)	20 (27.4%)
Values given as n (%). LMWH, low-molecular-weight heparin.				

### Primary and secondary outcomes

Bleeding events and death in the 30-day post-procedure follow-up period are shown in Table 3; there were no thromboembolic events documented. All bleeding events occurred in patients who received LMWH bridging. Of the four patients (2%) with clinically relevant bleeding, two patients (1%) had a MB and neither had a biopsy or polypectomy. One patient received bridging before and after and then had MB 12 days post-procedure and the other was bridged only after the procedure.

Two patients had CRNMB 10 and 18 days after the procedure: both received pre- and post-procedure bridging and neither had a biopsy or polypectomy. One of the patients who had CRNMB bleeding 18 days post-colonoscopy with no polypectomy and endoscopy with a gastrointestinal biopsy, both procedures happened on the same day. There was one death due to ventricular fibrillation occurring 13 days after a gastrointestinal endoscopy with biopsy; this patient did not receive any bridging. The overall rate of adverse events, including MB, CRNMB, and death, was 3%.

### LMWH bridging and bleeding outcomes

Prior to PSM, post-procedure LMWH was associated with the composite outcome of clinically relevant bleeding, comprising MB and CRNMB ( *P* = 0.034), regardless of the approach used before the procedure. Similar associations were observed in the pre-procedure only and combined pre- and post-procedure bridging groups. However, LMWH use was not significantly associated with the individual outcomes of CRNMB ( *P* = 0.20) or MB ( *P* = 0.18). Any LMWH use was not associated with MB ( *P* = 0.69), CRNMB ( *P* = 0.28), or at least one bleeding outcome ( *P* = 0.064). No covariates were significantly associated with bleeding or LMWH use.

In a stepwise multiple regression model, no variables entered the prediction model for at least one bleeding outcome, CRNMB or MB. Risk propensity scores were calculated using response variables from the AMBER trial. There were no significant differences in CRNMB or MB outcomes between the 66 propensity-matched patients (1:1 ratio) who received bridging and those who did not ( *P* = 0.99). Patients who only received pre-procedural bridging were not included in regression or propensity score analyses because this strategy is not commonly used in clinical practice and may indicate unusual or high-risk bleeding features identified during the endoscopy. In the overall AMBER cohort, undergoing a gastrointestinal procedure (OR 0.25; *P* = 0.002) and, specifically, a colonoscopy (OR 0.16; *P* = 0.012) were associated with a lower risk of bleeding compared with non-gastrointestinal procedures.

## Discussion

In this subanalysis of patients with an aortic MHV from the AMBER study who underwent elective gastrointestinal endoscopy, before PSM, we found that patients receiving preoperative and postoperative LMWH bridging were at higher risk of clinically relevant bleeding than those who were not bridged or who only received pre-procedure bridging, but absolute risks of MB and CRNMB were low. There were no significant differences in bleeding risk when performing polypectomies or biopsies, regardless of the bridging strategy used.

Although current guidelines include clear recommendations for periprocedural anticoagulation management in patients with atrial fibrillation, recommendations for MHV patients are variable. The International Digestive Endoscopy Network consensus recommends withholding warfarin based on bleeding risk, and recommends restricting the use of heparin bridging to patients at high thromboembolic risk 10 . In contrast, the 2022 American College of Gastroenterology-Canadian Association of Gastroenterology guideline considers bridging appropriate for all MHV patients, categorizing them as moderate thromboembolic risk 11 . Recommendations in both guidelines, however, are conditional based on a low quality of evidence given lack of relevant randomized trials or other prospective studies. Our findings align with the practice of withholding warfarin and suggest caution when using LMWH bridging due to an associated increased bleeding risk. We found that after PSM, patients who receive bridging do not appear to be at increased risk of bleeding; therefore, these data support safety of bridging patients who may be candidates for bridging based on particularly elevated thrombotic risk.

Previous studies examining risk of post-colonoscopy thromboembolic events in the context of warfarin interruption for gastrointestinal procedures in patients with a MHV are limited 12 13 . An analysis of the PAUSE study, which included 556 patients with atrial fibrillation who interrupted direct oral anticoagulants for elective gastrointestinal endoscopies reported a thromboembolic event rate of 0.7% (0.3%-1.8%), a bleeding rate of 2.5% (1.4%-4.2%), and a MB rate of 0.9% (0.4%-2.1%) 12 . Our finding of no thromboembolic events, our gastrointestinal bleeding rate of 2.08% and MB rate of 1.04% are similar to the PAUSE subanalysis. These results suggest that baseline bleeding risk of endoscopic procedures is low but may be amplified in patients receiving periprocedural LMWH bridging.

Bleeding risk associated with warfarin use during colonoscopies has also been studied in a 2-year audit of 5,593 cases with 1,657 polypectomies 13 . This study reported a 2.2% rate of polypectomy-associated bleeding and uninterrupted warfarin use was associated with an increase in post-polypectomy bleeding (OR 13.4; 95% CI 4.1–43.6) 13 . A multicenter prospective observational study, the AMBER trial, of 560 patients, 277 of whom continued antithrombotic therapies, found no significant increase in upper gastrointestinal bleeding risk compared with those who interrupted antithrombotics 6 . Our findings align with these trials because patients from the AMBER trial who underwent gastrointestinal procedures or colonoscopies were not found to be at a significant risk of any bleeding compared with those who had non-gastrointestinal procedures ( *P* = 0.0020, *P* = 0.0023) 6 .

The strengths of our analysis include its multicenter design, which encompasses a diverse range of bridging practices and patient types and detailed description of outcomes and treatment practices specific to gastrointestinal endoscopy in patients with aortic MHVs. Our analysis includes colonoscopies and upper gastrointestinal endoscopies, which are both common procedures requiring periprocedural anticoagulation management. As the largest cohort of MHV patients undergoing bridging for endoscopic procedures reported to date, these findings contribute important data with respect to different bridging practices and outcomes in this patient population.

There are several limitations of our study. First, the small cohort size limits the reliability of conclusions regarding the incidence of outcomes. Second, the retrospective nature of AMBER inherently affects the validity of our results, because with no manipulation of treatment groups, our conclusions reflect outcomes from earlier practices and do not directly inform changes in clinical practice. Third, these findings may be related to changes in pre- and post-procedure bridging, but no differences in when anticoagulation was resumed post-procedure. These limitations highlight the need for a study comparing bridging and no-bridging strategies in aortic MHV patients undergoing gastrointestinal endoscopies to better understand thromboembolic and bleeding risks.

## Conclusions

In conclusion, in patients with aortic MHVs having gastrointestinal endoscopic procedures, prior to PSM, post-procedure LMWH was associated with the composite outcome of clinically relevant bleeding, comprising MB and CRNMB ( *P* = 0.034), although not with MB or CRNMB individually, regardless of preprocedural strategy. There was no difference in CRB between groups after PSM. Procedures involving biopsies or polypectomies were not associated with an increased risk of bleeding and no thromboembolic events were observed during anticoagulant interruption. Larger prospective studies are needed to better define bleeding and thromboembolic risks, including in higher-risk populations, with and without periprocedural LMWH bridging.

## Supplementary Material

Supplementary Material Zusatzmaterial
