# Supplementary material for: Perioperative low molecular weight heparin bridging in aortic mechanical heart valve patients undergoing endoscopic procedures
Source: Endosc Int Open. 2026 Apr 17;14:a28407302. doi: 10.1055/a-2840-7302 (PMC13093117; doi:10.1055/a-2840-7302)
Supplement: Supplementary file 1 — Supplementary Material [file 10-1055-a-2840-7302_28472047.pdf]

Supplementary Table 1 Preprocedural anticoagulation characteristics.

Characteristic	Value
$CHADS_2$ Score (score, IQR)	2 (1–2)
Bridged with LMWH or UFH	
Yes — LMWH	110 (57.3%)
Yes — UFH	4 (2.1%)
No	82 (42.7%)
LMWH agent used	
Enoxaparin	77 (72.6%)
Tinzaparin	17 (16.0%)
Dalteparin	12 (11.3%)
Days between end of preprocedure LMWH and procedure (days, IQR)	1 (1-1)
VKA dosage (mg/week, IQR)	32.25 (23.4-45.0)
Days between last VKA dose and procedure (days, IQR)	6 (6-6)
Antiplatelet interruption	
Yes	68 (89.5%)
No	8 (10.5%)
Days between last antiplatelet dose and procedure (days, IQR)	4 (3-3.5)

Values given as n (%) or median (IQR). LMWH agent distribution reported among 106 patients who received LMWH bridging. Antiplatelet data reported among 76 patients on antiplatelet therapy.

$CHADS_2$, congestive heart failure; hypertension, age ≥ 75, diabetes, stroke/TIA (doubled); IQR, interquartile range; LMWH, low-molecular-weight heparin; TIA, transient ischemic attack; UFH, unfractionated heparin; VKA, vitamin K antagonist;

Supplementary Table 2 Postprocedural anticoagulation management.

Characteristic	Value
Full-dose LMWH continued after procedure	
Yes	68 (65.4%)
No	36 (34.6%)
Days between procedure and LMWH start (days, IQR)	1 (1-2)
Days between procedure and LMWH end (days, IQR)	5 (3-8)
VTE prophylaxis-dose LMWH after procedure	2 (1.1%)
Tinzaparin (units/day)	8,000
Dalteparin (units/day)	5,000
VKA first dose after procedure doubled	
Yes	64 (61.5%)
No	40 (38.5%)
Days post-procedure to VKA resumption (days, IQR)	0 (0-0)
Days post-procedure to antiplatelet resumption (days, IQR)	1 (0-1)

Values given as n (%) or median (IQR). Full-dose LMWH continuation and timing data reported among patients who received any post-procedure LMWH.
IQR, interquartile range; LMWH, low-molecular-weight heparin; VKA, vitamin K antagonist; VTE, venous thromboembolism.

Endosc Int Open | DOI 10.1055/a-2840-7302 | © The Author(s).

Supplementary Table 3 Standardized mean differences before and after propensity score matching.

Covariate	SMD before	SMD after	Reduction	Balanced*
Age	0.12	0.03	75%	✓
Sex (male)	0.08	0.02	75%	✓
Hypertension	0.18	0.04	78%	✓
Diabetes	0.06	0.01	83%	✓
Chronic kidney disease	0.10	0.05	50%	✓
Congestive heart failure	0.09	0.03	67%	✓
Coronary artery disease	0.21	0.06	71%	✓
Atrial fibrillation	0.17	0.05	71%	✓
Prior stroke or TIA	0.28	0.08	71%	✓
Prior venous thromboembolism	0.14	0.07	50%	✓
$CHADS_2$ score	0.23	0.06	74%	✓
Time since valve implantation	0.30	0.07	77%	✓

*Balance achieved defined as post-matching SMD < 0.10. All 12 covariates achieved adequate balance after matching (all post-matching SMDs: 0.01-0.08).

$CHADS_2$, congestive heart failure; hypertension, age ≥ 75, diabetes, stroke/TIA (doubled); SMD, standard mean difference; TIA, transient ischemic attack.
